# Assessment of PULP score in predicting 30-day perforated duodenal ulcer morbidity, and comparison of its performance with Boey and ASA, a retrospective study

**DOI:** 10.1016/j.amsu.2019.05.001

**Published:** 2019-05-10

**Authors:** Tamer Saafan, Walid El Ansari, Omer Al-Yahri, Ammar Eleter, Hisham Eljohary, Rashad Alfkey, Mustafa Hajjar, Ali Toffaha, Abdelrahman El Osta

**Affiliations:** a Department of General Surgery, Hamad General Hospital, Hamad Medical Corporation, Doha, 3050, Qatar; b Department of Surgery, Hamad General Hospital, Hamad Medical Corporation, Doha, 3050, Qatar; c College of Medicine, Qatar University, Doha, 2713, Qatar; d School of Health and Education, University of Skövde, Skövde, Sweden; e Department of Surgery, AlWakra Hospital, AlWakra, Qatar

**Keywords:** Perforated peptic ulcer score, PULP, Perforated duodenal ulcer, Boey, ASA, Perforated peptic ulcer, PDU, Perforated peptic ulcer, PPU, perforated peptic ulcer, M&M, mortality and morbidity, PULP, peptic ulcer perforation score

## Abstract

**Background:**

*/aim*: Scores commonly employed to risk stratify perforated peptic ulcer patients include ASA (American Society of Anesthesiologists), Boey and peptic ulcer perforation score (PULP). However, few studies assessed and compared the accuracy indices of these three scores in predicting post PPU repair 30-day morbidity. We assessed accuracy indices of PULP, and compared them to Boey and ASA in predicting post perforated duodenal (PDU) ulcer repair 30-day morbidity.

**Methods:**

Retrospective chart review of all PDU patients (perforated duodenal ulcers only) at the largest two hospitals in Qatar (N = 152). Data included demographic, clinical, laboratory, operative, and post repair 30-day morbidity. Area under the Curve (AUC), sensitivity and specificity were computed for each of the 3 scores. Multivariate logistic regression assessed the accuracy indices of each score.

**Results:**

All patients were males (*M* age 37.41 years). Post PDU repair 30-day morbidity was 10.5% (16 morbidities). Older age, higher ASA (≥3), Boey (≥1) or PULP (≥8) scores, shock on admission and preoperative comorbidities; and conversely, lower hemoglobin and albumin were all positively significantly associated with higher post PDU 30-day morbidity. PULP displayed the largest AUC (72%), and was the only score to significantly predict 30-day morbidity. The current study is the first to report the sensitivity and specificity of these three scores for post PDU repair 30-day morbidity; and first to assess accuracy indices for PULP in predicting post PDU repair 30-day morbidity.

**Conclusion:**

PULP score had the largest AUC and was the only score to significantly predict post PDU repair 30-day morbidity.

## Highlights

• The current study is the first to report the sensitivity and specificity of the three scoring systems (ASA, Boey and PULP) for post perforated duodenal ulcer repair 30-day morbidity.
• It is also the first study to assess accuracy indices for PULP in predicting post perforated duodenal ulcer repair 30-day morbidity.
• Compared with ASA and Boey, PULP score had the largest area under the curve and was the only score to significantly predict post perforated duodenal ulcer repair 30-day morbidity.

## 1 Introduction

Perforation is the second most common complication of peptic ulcer [1], complicating 2–10% of peptic ulcers [2], where mortality and morbidity (M&M) may reach 25% and 50% respectively [3]. Several scoring systems have been proposed for the prediction of 30**-**day M&M of perforated peptic ulcer (PPU) in order to risk stratify patients subject to their anticipated complications, and accordingly direct the required attention to high-risk patients.

Scoring systems most commonly used include ASA (American Society of Anesthesiologists) [4], Boey [5] and peptic ulcer perforation score (PULP) [4]. Each comprises 3–11 demographic, clinical and biochemical variables that consider only pre-operative, or include pre/intra –operative and laboratory findings [6]. Clinical scoring systems need good diagnostic accuracy in order to risk stratify patients correctly.

The current literature however has some gaps. First, research on PULP is rare, with inconsistent accuracy indices [4,7,8]. Second, some studies used/assessed one scoring system only [[9], [10], [11], [12], [13], [14]] and did not compare the different scores commonly used. Third, comparisons of several PPU scores are sparse [4], where only 3 studies compared 3 PPU scores [4,8,15] and only 4 studies compared 4 PPU scores [7,[16], [17], [18]]. Fourth, in terms of outcomes, studies of PPU scores assessed mainly mortality [4,7,8,18] rather than morbidity [15,16], although post repair PPU morbidities are more common and serious (bleeding, perforation, obstruction) [1]. In addition, the two studies [15,16] that assessed morbidity had limitations. First, both computed the Area under the Curve (AUC) only, with no report of sensitivity and specificity indices [19]. The second limitation is that AUC was computed for individual morbidities (septic shock and ICU admission) [16], with no accuracy indices provided for overall morbidity that reflect a patient's overall risk. Fifth, studies that compared PPU scores employed different case mixes that included repair for perforated gastric and duodenal ulcers [4,7,8,15,16,18], where no study compared the scores based on repair of only gastric ulcers or only duodenal ulcers, despite that perforated gastric ulcers have more serious outcomes [20].

In order to bridge these gaps, this retrospective study assessed the 30-day morbidity of PDU across four years; evaluated PULP's ability to predict post 30-day post PDU repair morbidity; and compared PULP's performance with Boey and ASA in predicting post repair 30-day morbidity of only perforated duodenal (not gastric) ulcers. To best of our knowledge, the current study is the first globally to examine PULP in predicting post PDU repair 30-day morbidity, and first to compare the accuracy indices of PULP, Boey and ASA in predicting morbidity among only PDU patients. The objectives were to:

• Measure the post PDU repair 30-day morbidity, its types, and patient characteristics;
• Assess the accuracy (AUC, sensitivity, specificity) of PULP score in predicting post PDU repair 30-day morbidity; and,
• Compare the accuracy of Boey and ASA and Boey in predicting post PDU repair 30-day morbidity.

## 2 Methods

### 2.1 Settings, ethics and procedures

This retrospective study was undertaken at the two largest tertiary care centers in Qatar, at Hamad General (Doha) and Alwakra (AlWakra city) Hospitals, both part of Hamad Medical Corporation (HMC, equivalent of Ministry of Health). HMC's Medical Research Centre approved the study (#17081/17). This study is registered in Researchregistry.com, and was written in line with the STROCSS statement [21].Using the hospital's administrative electronic database, we reviewed charts of all patients diagnosed and operated for perforated duodenal ulcers (January 2014–December 2017). Data included demographic, clinical, laboratory, operative, postoperative information and complications within 30 days. Patients <14 years old or with perforated other organs (e.g. gastric ulcer or intestinal perforation excluding duodenum) and were excluded.

### 2.2 ER triage system

All patients presenting to the ER with severe abdominal pain are seen immediately by ER doctor. Where perforated viscus is suspected, the patient is resuscitated in an ER high dependency unit/ICU, upright CXR may be done to detect air under diaphragm, and patient is seen by a surgeon within a maximum of 30 min. Once diagnosis of perforated viscus is confirmed, patient is operated within 60–90 min. Post-operatively, sick patients may be shifted to ICU for further management.

### 2.3 Main outcome measure

The main outcome (primary end point) was post PDU repair 30-day morbidity.

### 2.4 Definitions

*Shock on admission*: for PULP, defined as blood pressure <100 mm Hg and heart rate >100 beats/min [8]. For Boey, defined as only blood pressure <100 mm Hg [5].

*Perforation* > *24 h*: In PULP, it is time interval from perforation (onset of or aggravation of symptoms) until admission to hospital [8]. In Boey, it is the time interval from perforation until surgery [5].

*Perforated peptic ulcer* (PPU): includes both perforated gastric and perforated duodenal ulcers.

*Perforated duodenal ulcer* (PDU): includes only perforated duodenal ulcers (focus of current study).

### 2.5 Statistical analysis

Statistical analyses were done using statistical packages SPSS 22.0 (SPSS Inc. Chicago, IL) and Epi Info 2000 (Centers for Disease Control and Prevention, Atlanta, GA). A two-sided P value < 0.05 was considered statistically significant. Qualitative and quantitative data values were expressed as frequency with percentage and mean ± SD with median and range. Descriptive statistics summarized participants’ demographic, medical history and clinical characteristics along with post-surgical complications. Data analysis assessed the post PDU repair 30-day morbidity and accuracy of the three scores (PULP, ASA, Boey) in predicting post PDU repair 30-day morbidity. Hence, the sensitivity and specificity values of these scores were computed and compared. A receiver operating characteristic (ROC) curve was calculated using potential predictors (as determined via univariate and multivariate logistic regression) to derive best cut-off values and assess model discrimination and predictive accuracy. ROC curves summarized the accuracy of predictions in a visually comprehensive way.

Associations between two or more qualitative variables were assessed using Chi-square and Fisher exact tests. Quantitative data compared between two independent groups were analyzed using unpaired ‘t’ and Mann-Whitney U tests. Univariate and multivariate logistic regression methods assessed the predictive values of each predictor or risk factor (clinical signs and symptoms, PULP, ASA, Boey) for post PDU repair 30-day morbidity. For multivariate regression models, variables were considered if significant at P < 0.10 in univariate analysis or if clinically important. Logistic regression analyses reported odds ratio (OR) with 95% confidence intervals (CIs).

### 2.6 Three clinical scoring systems

For each patient, three clinical scores were computed:

Boey, calculated by presence of shock, delay from admission to surgery >24 h, and high degree of co-morbidity, e.g. chronic obstructive pulmonary disease, heart failure, active cancer [5].

ASA, based on patients’ pre-existing co-morbidity, considers the present clinical condition at admission and is graded 1–5 increasingly indicating a healthy person, mild systemic disease, severe systemic disease, severe systemic disease that is a constant threat to life and a moribund person not expected to survive without operation [7].

PULP is a seven-variable score (range = 0–18), based on age >65 years, liver failure, AIDS/active cancer, concomitant use of steroids, shock on admission, time from admission to surgery >24 h, serum creatinine >130 (μmol/l) and ASA score [8].

## 3 Results

Data of all PDU patients were included (N = 152). Post PDU repair 30-day morbidity and mortality were 10.5% (16 morbidities) and 0.7% (1 mortality) respectively. Most patients (92%) had laparoscopic repair of PDU, 6% had laparoscopic converted to open repair and 2% had open repair. We observed only one mortality; hence mortality was excluded from further analysis. Table 1 shows that the most common postoperative complications included abdominal collection (8 cases), lung complications and septic shock (7 and 5 cases respectively). Surgical site infection, DVT and ileus were less common.

**Table 1:** Postoperative complications following repair of perforated duodenal ulcer.

30-day Complication	Frequency (n)
Abdominal Collection	8
Pleural effusion/Pneumonia	7
Surgical site infection	3
Septic shock	5
DVT	1
Ileus	1
Total Morbidity	16
Mortality	1

DVT: deep vein thrombosis.

Table 2 shows the characteristics of PDU patients by 30-day morbidity for continuous variables. All patients were males, and mean age was 37.41 years (median = 35 years). Older age and higher ASA, Boey or PULP scores; and conversely, lower levels of hemoglobin and albumin were all positively significantly associated with higher 30-day morbidity. Creatinine and WBC levels were both not associated with post repair 30-day morbidity.

**Table 2:** Characteristics of PDU patients by 30-day morbidity (continuous variables).

Variable	All Sample	30-day morbidity		P
		Yes	No	
Demography				
Gender (M:F)	151:0	–	–	–
Age (years)	37.41 ± 12.6	44.94 ± 14.30	36.51 ± 12.16	0.002
Chemistry				
WBC (uL)	13.43 ± 5.83	10.89 ± 6.15	13.73 ± 5.74	0.089
Hemoglobin (gm/dl)	15.00 ± 2.12	13.61 ± 2.62	15.17 ± 2	0.003
Creatinine (umol/L)	93.92 ± 52.00	153.31 ± 127.01	87 ± 27.65	0.063
Albumin (gm/L)	37.80 ± 6.05	31.81 ± 9.70	38.52 ± 5.06	0.01
Score				
ASA	2.14 ± 0.69	2.56 ± 0.73	2.10 ± 0.67	0.001
Boey	0.64 ± 0.63	1.06 ± 0.78	0.59 ± 0.59	0.001
PULP	2.21 ± 1.97	3.81 ± 2.93	2.02 ± 1.74	0.016

All cell values represent mean ± standard deviation; M: F male: female; —: not applicable.

Table 3 depicts the characteristics of PDU patients by 30-day morbidity for categorical variables. Shock on admission and preoperative comorbidities were both significantly associated with 30-day morbidity. Likewise, ASA level ≥3, Boey ≥1 and PULP ≥8 were significantly associated with post repair 30-day morbidity. Conversely, perforation on admission >24 h, malignancy and liver cirrhosis were not associated with post repair 30-day morbidity.

**Table 3:** Characteristics of PDU patients by 30-day morbidity (categorical variables).

Variable	n	30-day morbidity		P
		Yes %	No %	
Perforation on admission >24 h a	73	12.2	87.8	0.7
Shock on admission b	3	66.7	33.3	0.03
Preoperative comorbidities	13	38.5	61.5	0.001
Malignancy	2	0	100	1
Liver cirrhosis	1	100	0	0.112
ASA Level				0.004
1	23	4.30	95.7	
2	86	7.0	93.0	
3	39	20.5	79.5	
4	3	33.3	66.7	
5	0	0	0	
Boey Level				0.001
0	65	6.20	93.8	0.001
1	76	9.20	90.8	
2	9	55.5	44.5	
3	1	0	100	
Pulp Level				
0-7	140	8.57	91.43	
8-18	11	36.36	63.64	

a We used the PULP's definition. Although Boey's definition differs from PULP, however, as most of our patients were operated within 1–2 h from admission to emergency room, such difference in definitions did not influence the current study.

b We used the (PULP's) definition as we had only 3 patients presenting with both blood pressure < 100 mm Hg and pulse > 100, therefore, even these three patients fitted both (Boey and PULP) definitions.

**Fig. 1:**
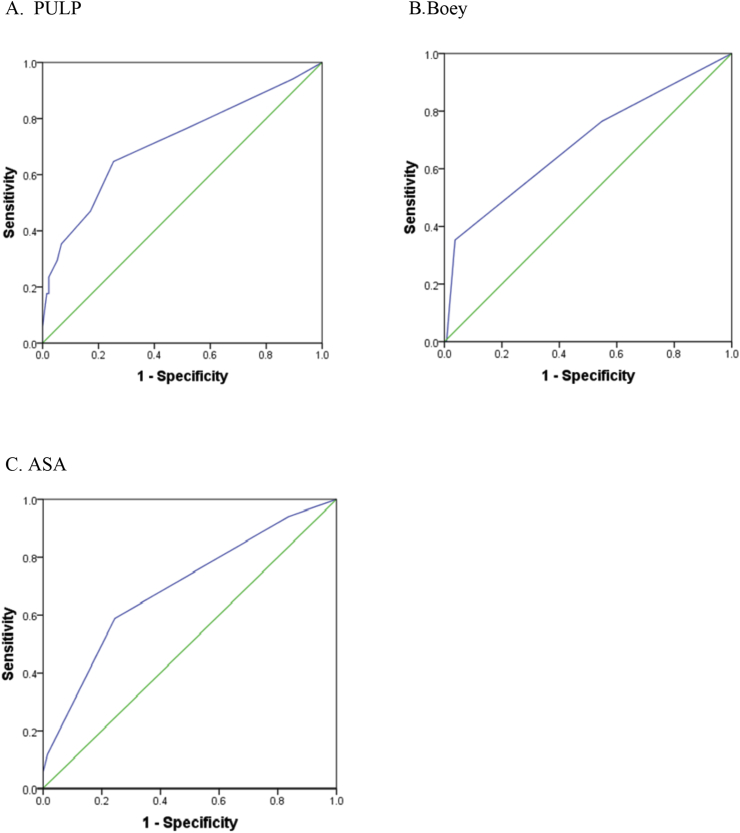
ROC curves for PULP, Boey and ASA showing the area under the curve.

In order to assess PULP's predictions of post PDU repair 30-day morbidity, ROC analysis shows that AUC was 72% at cutoff value of ≥3 (Fig. 1A, Table 4), with sensitivity and specificity of 64.71% and 74.63%, respectively.

**Table 4:** 30-day morbidity optimal cut-off and accuracy indices of three scoring systems^a^.

Variable	AUC (95% CI)	P	Cutoff	Sensitivity (95% CI)	Specificity (95% CI)
PULP	0.72 (0.57–0.86)	0.004	≥3	64.71 (41.30–82.69)	74.63(66.64–81.24)
ASA	0.69 (0.55–0.83)	0.009	≥3	58.82 (36.01–78.39)	75.56 (67.66–82.03)
Boey	0.69 (0.54–0.84)	0.011	≥1	76.47 (52.74–90.45)	45.19 (37.04–53.60)

a Based on receiver operating characteristics (ROC) curve analysis for 30-day morbidity; AUC: Area under the curve; CI: confidence interval.

PULP's accuracy indices were compared with those of ASA and Boey (Fig. 1, Table 4) using ROC analysis. Fig. 1 depicts that PULP had largest AUC (72%, P = 0.009), while ASA and Boey exhibited similar slightly less AUCs (both 69%, P = 0.009 and 0.01 respectively). Table 4 shows that Boey exhibited the highest sensitivity (76.47% at cutoff value ≥ 1), while ASA displayed the highest specificity (75.56% at cutoff value ≥ 3).

Finally, using multivariable logistic regression analysis, PULP was the only significant score in predicting the 30-day morbidity following PDU repair, with odds ratio 5.39, 95% confidence interval 1.85–15.69, P = 0.002.

## 4 Discussion

Several studies assessed PULP score in predicting 30-day post PPU repair mortality [4,7,8,18,22]. To the best of our knowledge, the current study is first to examine PULP in predicting post PDU repair 30-day morbidity, and first to compare the accuracy indices of PULP, Boey and ASA in predicting morbidity among only PDU patients. Our post PDU repair 30-day complications (17 patients) comprised 16 morbidities and 1 mortality. This mortality was excluded from further analysis, and the current analysis represents the post PDU repair 30-day morbidity only.

The 30-day post PDU repair mortality (0.7%) of the current study was lower than the PPU repair mortality reported in Thailand (9%), Singapore (7.2%), Norway (16.3%) and Denmark (17%) [4,7,15,16] (Table 5). Likewise, the 30-day post PDU repair morbidity (10.5%) we observed was lower than that of PPU repair morbidity of Singapore (11.4%), Turkey (24.2%), Thailand (30%) and Norway (52%) [4,7,15,18]. The current study's low morbidity and mortality might be due to: a) rapid triage, all patients were operated within 2 h of ER admission; b) laparoscopic PDU repair, associated with lower M&M [23,24]; c) lower mean patient age than other studies [25]; d) no females, female gender may be more associated with PPU post-operative mortality [25]; and, e) examining only perforated duodenal ulcers (Table 5), in contrast with other studies that examined combinations of both perforated duodenal and gastric ulcers. Perforated gastric ulcers are associated with higher mortality [20].

**Table 5:** Cut-off and diagnostic accuracy indices of scoring systems for predicting 30-day morbidity after PPU repair.

Study^a^	Complication	G/D (%)	N (%)	Score used	Cutoff	Sensitivity	Specificity	AUC%
Current Study 2018 Qatar (N = 151)	Morbidity	0/100	16(10.5)	ASA	≥3	58.82	75.56	69
				Boey	≥1	76.47	45.19	69
				PULP	≥3	64.71	74.63	72
Lohsiriwat 2008 Thailand [15] (N = 152)	Morbidity	86/14	46(30)	ASA	**—**	**—**	**—**	80
				Boey	**—**	**—**	**—**	80
Buck 2011 Denmark [16] (N = 117)	Septic shock	35.9/64.1	30(25.6)	ASA	≥3	**—**	**—**	67
				Boey	≥2	**—**	**—**	72
	ICU admission		49(41.9)	ASA	≥3	**—**	**—**	69
				Boey	≥2	**—**	**—**	64
Thorson 2014 Norway [4] (N = 172)	Morbidity	65.1/34.9	89(52)	**—**	**—**	**—**	**—**	**—**
Anbalakan 2015 Singapore [7] (N = 332)	Intraabdominal collection	56.9/40.4	27	**—**	**—**	**—**	**—**	**—**
	Leakage		7	**—**	**—**	**—**	**—**	**—**
	Reoperation		4	**—**	**—**	**—**	**—**	**—**
	Morbidity		38(11.4)	**—**	**—**	**—**	**—**	**—**
Menekse 2015 Turkey [18] (N = 227)	Morbidity	**—**	55(24.2)		**—**	**—**	**—**	**—**

a Only the first author is cited for space consideration; G/D: Case mix, percentage of perforated gastric ulcer to perforated duodenal ulcer; N: number of patients; —: not reported.

As for patient characteristics, in terms of demography, our sample's mean age (37.41 years) was lower than the means reported by others (e.g. 50.6 years [18], 48 years [15]), supporting that PDU occurs at younger ages [26], and the association of older age with poorer prognosis and increased post-operative morbidity [8,14,27]. In terms of gender, a study of 99 PDU patients had 98 male and only one female patient [28]. We agree; our sample comprised no females, probably because PDU is far more in common in males. In addition, Qatar's population structure male: female ratio is 3:1 due to the large numbers of immigrant single males [29].

We observed that pre-operative co-morbidities, shock and lower albumin were significantly associated with post PDU repair 30-day morbidity; while perforation on admission >24 h, liver cirrhosis and malignancy were not significantly associated with morbidity (Table 3), in agreement with others [14,18,25,27]. A novel finding not previously reported is that lower hemoglobin was significantly associated with 30-day morbidity (although this lower level was still within the normal range); others found no such association [18]. Conversely, in the present study, both creatinine and WBC levels were not significantly associated with 30-day morbidity. While our findings contrast with that high creatinine was significantly associated with 30-day morbidity [18,25], for WBCs, we support the non-significant association of WBCs with 30-day morbidity [18]. For deeper understandings of the associations of such variables with 30-day morbidity following repair of different types of perforated ulcers, studies with larger patient samples, separation of the outcomes of morbidity and mortality, as well as separation of perforated gastric and duodenal ulcers are required.

PULP has been assessed mainly for mortality, with inconsistent results e.g. AUC 83% at cut-off > 7 for high risk patients [8]; AUC 79% at cut-off > 6, 92.9% sensitivity and 58.3% specificity [4]; and AUC 75% at cut-off >7 [7]. Although only one study assessed PULP for post PPU repair morbidity [22], this study did not specify the time period of the morbidity (whether 30-day or longer term), employed case mixes that included repair for perforated gastric and perforated duodenal ulcers, and did not report AUC, cut-off value, sensitivity and specificity. The current study examined PULP in predicting 30-day post repair morbidity of only perforated duodenal ulcers, where the AUC was 72% at cutoff ≥3, with 64.71% sensitivity and 74.63% specificity (Fig. 1A, Table 4). A point to note is that PULP was developed for predicting mortality [4,7,8,18,22] rather than morbidity. However, the accuracy findings of the current study are novel in that they suggest that PULP’s performance in predicting 30-day post PDU repair morbidity (AUC 72%) is certainly close to the findings reported by Anbalakan et al. [7] (AUC 75%) and Thorsen et al. [4] (AUC 79%) for PULP's accuracy in predicting 30-day post PPU repair mortality.

Comparing PULP's accuracy with ASA and Boey in predicting post PDU repair 30-day morbidity shows that higher Boey, ASA and PULP scores were all significantly associated with post PDU repair 30-day morbidity (Table 2, Table 3). As Boey increased from 0 to 1 to 2, the 30-day morbidity increased in a positive ‘stepladder’ fashion from 6.2 to 9.2–55.5%, in agreement with others [4,7,16]. Likewise, higher ASA was also associated with ‘stepladder’ increase of 30-day morbidity, supporting the significant association between higher ASA and post PPU repair 30-day morbidity reported by others [7,16]. As for PULP, this study is first to report a significant positive relationship between PULP and 30-day PDU morbidity, where a score >7 significantly increased the morbidity percentage by almost four folds (Table 3).

In terms of cut-offs, no study computed Boey or ASA cut-offs for post PDU repair 30-day morbidity (Table 5); we found that ASA ≥ 3 and Boey ≥ 1 best predicted post PDU repair 30-day morbidity, in partial agreement with others [16] who observed similar ASA, but higher Boey (≥2) cut-offs for post PPU repair 30-day morbidity (Table 5). Likewise, no study inspected PULP's association with the 30-day morbidity for only PDU; the current study observed that higher PULP was positively significantly associated with 30-day PDU morbidity (Table 2, Table 3). Our PULP cutoff of ≥3 is the first reported for 30-day morbidity prediction post PDU repair. Further research for Boey, ASA and PULP cut-offs that best predict 30-day post PDU morbidities is needed.

An ideal scoring system needs to be an effective diagnostic indicator for identifying complex cases, and the AUC reflects a score's accuracy, its discriminatory ability to correctly classify patients, where 70–80% AUC is considered fair accuracy [30]. Other studies [15,16] reported AUC for ASA and Boey in predicting 30-day PPU morbidity. For ASA, our 69% AUC agreed with the reports of others of 67% [17] and 80% [16] AUC; for Boey, our 69% AUC also agreed with other research that found 72% [16] and 80% [15] AUC (Table 5). The disparities between our AUC and others' may be explained by different cut-off values for each score, different patient characteristics (e.g. mean age, percentage of comorbidities), and different proportions of gastric to duodenal perforations. In terms of sensitivity and specificity, the current study observed that Boey had highest sensitivity (76.47%), and ASA the highest specificity (75.56%). PULP exhibited the second highest sensitivity (64.71%) and specificity (74.63%), but had the highest AUC (72%) (Table 5). We are unable to precisely compare our findings with others as no studies examined the sensitivity and specificity of these three scores in predicting post repair morbidity (Table 5). Finally, the multivariable regression analysis showed that PULP was the only significant factor in predicting post PDU 30-day morbidity.

This study has limitations. The current study observed only one 30-day post PDU repair mortality, hence no further analysis was undertaken for mortality. Our patients were young (mean 37.41 years), consequently only 8.6% had co-morbidities compared with other studies (73%, 68%, and 16.2% co-morbidities) [7,8,16]. We did not examine other scoring systems (e.g. APACHE II [17], SAPS II [17], MPM [17], Jabalpur [30], MPI [15]) as these studies did not include any of the three systems (ASA, Boey or PULP) under examination and hence were out of the scope of the current study. Likewise, we did not examine other potential biomarkers (platelet to lymphocyte ratio, neutrophil to lymphocyte ratio) for predicting mortality in peptic ulcer perforation [31].

## 5 Conclusions

The current study is the first to simultaneously examine Boey, ASA and PULP scores for PDU only, and assess the association of PULP with post PDU repair 30-day morbidity. Higher PULP, Boey and ASA scores were all positively significantly associated with post PDU repair 30-day morbidity. PULP had the largest AUC, and was the only significant score of the three we examined to predict 30-day morbidity. Nonetheless, PULP's AUC was 72% reflecting fair accuracy. The literature exhibits deficiencies and inconsistencies in terms of the cut-off values, AUCs, sensitivities and specificities of these three scoring systems in predicting morbidity/mortality. Further prospective studies with larger patient samples and separation of the outcomes of morbidity and mortality, as well as separation of perforated gastric and duodenal ulcers are required in order to assess the efficacy of PULP score as a predictor of complications, and for better comparisons of PULP with other scoring systems.

### Provenance and peer review

Not commissioned, externally peer reviewed.

## Conflicts of interest

This work was not supported by any funding, and no funding body was involved with the design of the study; collection, analysis, and interpretation of data; or with any writing for this manuscript. Open Access funding provided by the Qatar National Library.

## Sources of funding

None.

## Ethics approval and consent to participate

The Medical Research Centre at Hamad Medical Corporation approved the study (IRB, Proposal #17081/17).

## Ethical statement

The study was approved by ethics committee and performed in accordance with the ethical standards laid down in the Declaration of Helsinki and its later amendments.

## Research registration unique identifying number (UIN)

Researchregistry4687.

## ISRCTN

Not applicable.

## Author contribution

Tamer Saafan initiated the project implementation and strategy, collected the patient data, drafted parts of the manuscript and assisted with the editing. Walid El Ansari initiated the project implementation and strategy, undertook data assessment, drafted the manuscript, and edited the manuscript. Omer Al-Yahri collected the patient data and assisted with the editing. Hisham Eljohary collected the patient data and assisted with the editing. Rashad Alfkey collected the patient data and assisted with the editing. Abdelrahman El Osta collected the patient data and assisted with the editing. Mustafa Hajjar collected the patient data and assisted with the editing. Ali Toffaha collected the patient data and assisted with the editing. Abdelrahman El Osta collected the patient data and assisted with the editing. All authors have read and approved the final manuscript.

## Guarantor

Prof. Walid El Ansari.
